# The complete chloroplast genome of *Ardisia brevicaulis* Diels 1900, one traditional medicinal plant in southern China

**DOI:** 10.1080/23802359.2022.2161326

**Published:** 2023-01-08

**Authors:** Zhiwei Wang, Youqiong Hu, Shenghua Wei

**Affiliations:** College of Pharmacy, Germplasm Resources Development and Cultivation Technology Research Center of Guizhou Genuine Medicinal Materials Ganoderma lucidum, Guizhou University of Traditional Chinese Medicine, Guiyang, China

**Keywords:** *Ardisia*, chloroplast genome, medicinal plant, phylogenetic analysis

## Abstract

The complete chloroplast (cp) genome of *Ardisia brevicaulis* Diels 1900, one traditionally medicinal plant usually used in southern China, was first assembled and reported in this study. The genome size is 156,742 bp (37.1% GC content), containing a large single-copy (LSC) region of 86,329 bp, a small single-copy (SSC) region of 18,417 bp, and a pair of inverted repeat regions (IRs) of 25,998 bp. 134 genes (89 protein-coding, 37 tRNA, and 8 rRNA genes) are annotated in the whole cp genome, including 115 unique genes (81 protein-coding, 30 tRNA, and 4 rRNA genes). Phylogenetic analysis showed that *A. brevicaulis* is closely related to *A. primulifolia* and *A. villosa*, indicating their close phylogenetic relationship. The cp genome of *A. brevicaulis* could provide valuable genomic information for the phylogeny, molecular identification and discovery of new medicinal plant resources in *Ardisia* Swartz 1788.

*Ardisia brevicaulis* Diels 1900, one species of *Ardisia* Swartz 1788 from the family Primulaceae Batsch ex Borkh.1797 (Chen and Pipoly 1996; APG IV 2016), is an important medicinal plant species widely distributed in southern China (González de Mejía and Ramírez-Mares 2011). The root of *A. brevicaulis* is usually used as a traditional chinese medicine for menstrual disorders, injuries and rheumatic pains (González de Mejía and Ramírez-Mares 2011; Li et al. 2010). Moreover, chemical and pharmacological studies showed that abundant natural products isolated from the root of *A. brevicaulis* have the potential biological activity of anti-tumor effect (Chen et al. 2011; Zhu et al. 2012). In particular, Ardisiphenol D, a major active resorcinol derivative among all the products, was shown to have potent inhibitory activity against cancer cells both *in vitro* and *in vivo* (Zhao et al. 2014). Due to the large number of species (65 species in China and about 400 to 500 species worldwide) in *Ardisia* and the similar morphology of *Ardisia* species (Chen and Pipoly 1996), the morphological classification and identification within the genus are difficult (Julius et al. 2021). Molecular phylogeny based on nrITS better revealed that *Ardisia* is not monophyletic as currently circumscribed, but left the phylogenetic relationship among many species of *Ardisia* unresolved, including the relationship between *A. brevicaulis* and its related species (Julius et al. 2021). In molecular phylogenetics, the chloroplast genomes can provide valuable sources of phylogenetic information because of their relatively stable genome structure and high evolutionary rates (Ravi et al. 2008). In this study, we first reported the complete chloroplast (cp) genome of *A. brevicaulis* and constructed the phylogenetic relationship with other *Ardisia* species. The results could be helpful for the phylogeny, molecular identification and discovery of new medicinal plant resources in *Ardisia*.

## Materials

**Figure 1.**
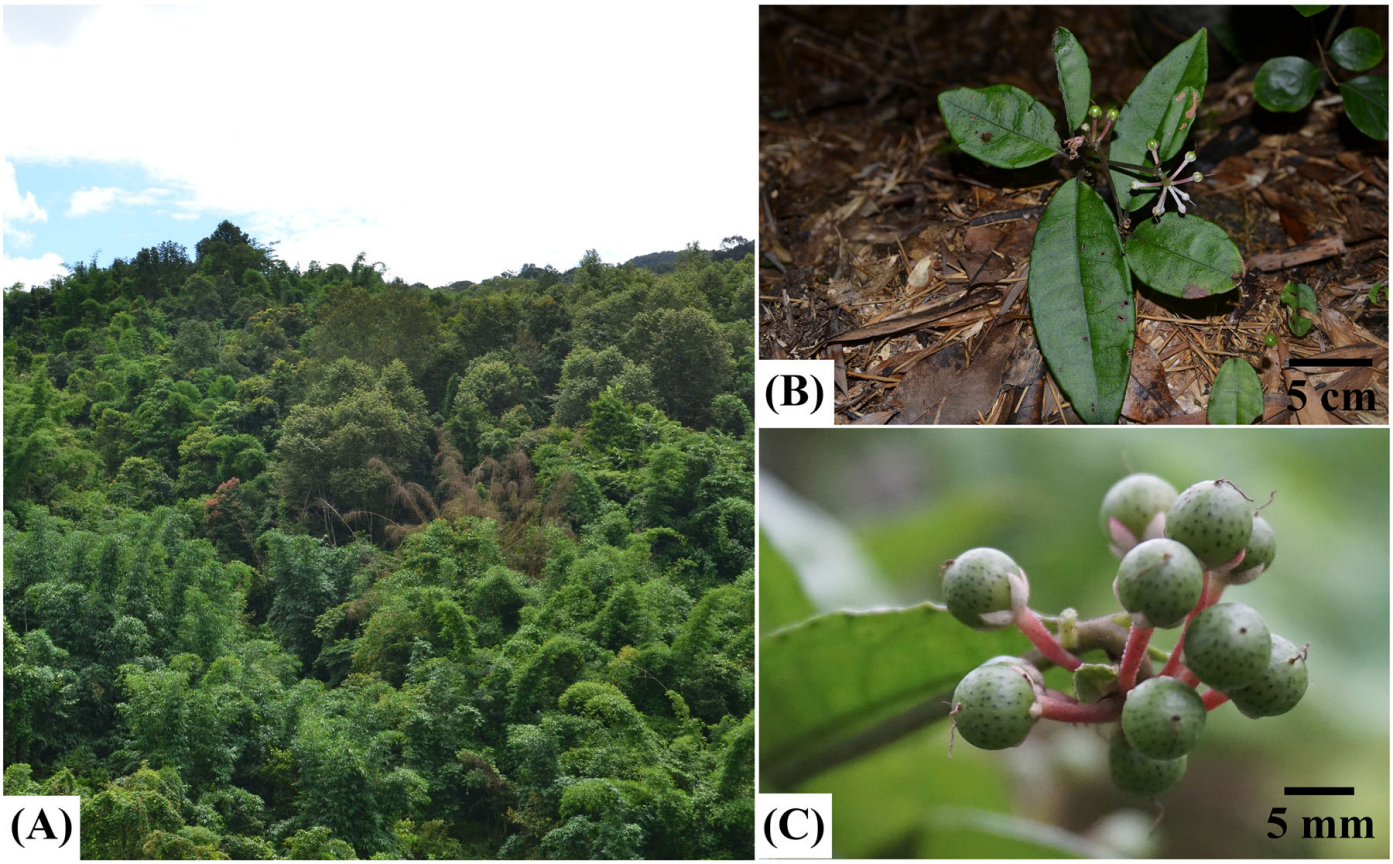
Images of the living plants of *Ardisia brevicaulis* and its growing environment. A.growing environment, B. living plants, C. fruits. Photos of species reference image were taken by the authors.

The sample of *A. brevicaulis* was collected from a wild population (108°35′12.12″E, 25°34′52.32 N; 910 m) in Congjiang County, Guizhou Province, China. It is a subshrub (10–15 cm) usually growing in dark and humid places under mixed forests (Figure 1; Chen and Pipoly 1996). It is characterized by its narrowly ovate to elliptic or suboblong leaf blade (7–18 × 2.5–6 cm) with an entire margin and indistinct marginal glandular dots, terminal umbellate inflorescences, globose punctate fruits (*c*. 6 mm in diameter) with usually purplish red persistent calyx and fruiting stalk (Figure 1; Chen and Pipoly 1996). The specimen of *A. brevicaulis* in this study was deposited at the herbarium of Guizhou University of Traditional Chinese Medicine (Chenggang Hu, Email: 2274547063@qq.com) under voucher number WZW20210304.

## Methods

Genomic DNA was extracted from fresh leaves of *A. brevicaulis* using Tiangen DNA Extraction Kit (DP305, Tiangen, Beijing, China) following the instructions of the kit. Purified genomic DNA was sheared into fragments (*c*. 350 bp) to construct a paired-end (PE) library. PE reads (*c*. 150 bp) were generated based on Illumina NovaSeq 6000 (Beijing Novogene Technology Co. Ltd). In total, 1.14 Gb clean data was retained after removing low quality reads and adaptor sequences. Based on the clean data, we assembled the cp genome of *A. brevicaulis* using GetOrganelle v1.7.5 (Jin et al. 2020), and annotated it *via* PGA (Qu et al. 2019), with further manual check and adjustment in Geneious v10.2.2 (Kearse et al. 2012). Annotated cp genome of *A. brevicaulis* was deposited to NCBI under the accession number ON208988.

The phylogenetic relationship between *A. brevicaulis* and other *Ardisia* species was inferred based on the whole cp genome sequences, with *Tapeinosperma multiflorum* and *T. netor* as outgroups (Hou et al. 2019; Shi and Liu 2019; Yan et al. 2019). Sequences in this study were aligned using MAFFT v7.397 (Katoh and Standley 2013). A maximum-likelihood (ML) tree was reconstructed in IQ-TREE v2.2.0 (Minh et al. 2020) using the best-fit substitution model (K3Pu + F+R2) chosen by ModelFinder (Kalyaanamoorthy et al. 2017). Branch supports were tested using SH-like approximate likelihood ratio (SH-aLRT) and Ultrafast bootstrap (UFBoot) with 10,000 bootstrap replicates.

## Results

**Figure 2.**
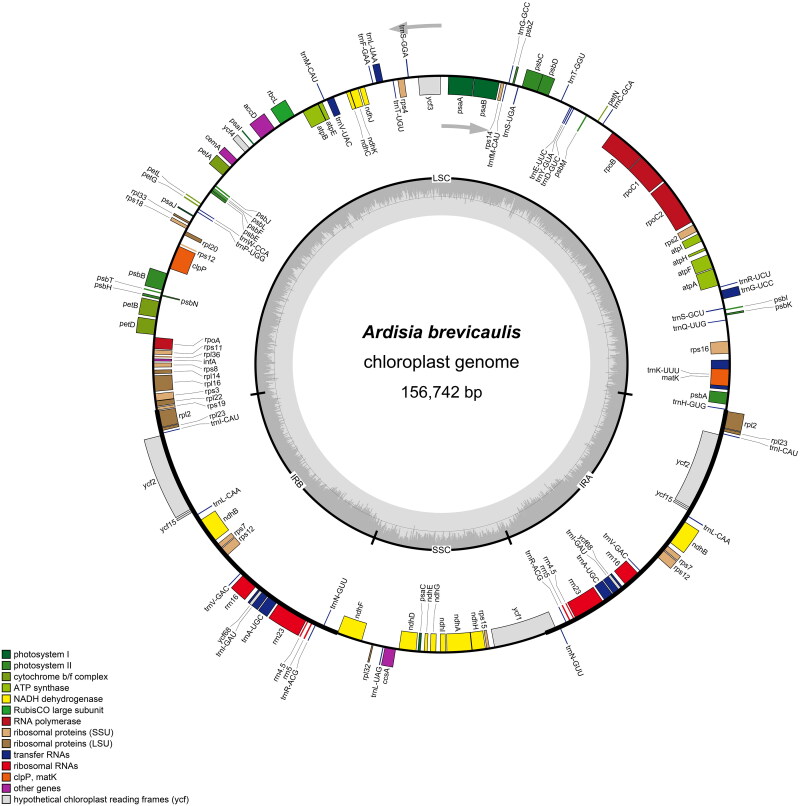
Circular map of the chloroplast genome of *Ardisia brevicaulis*. Genes drawn inside the circle are transcribed clockwise, drawn outside counter clockwise.

**Figure 3.**
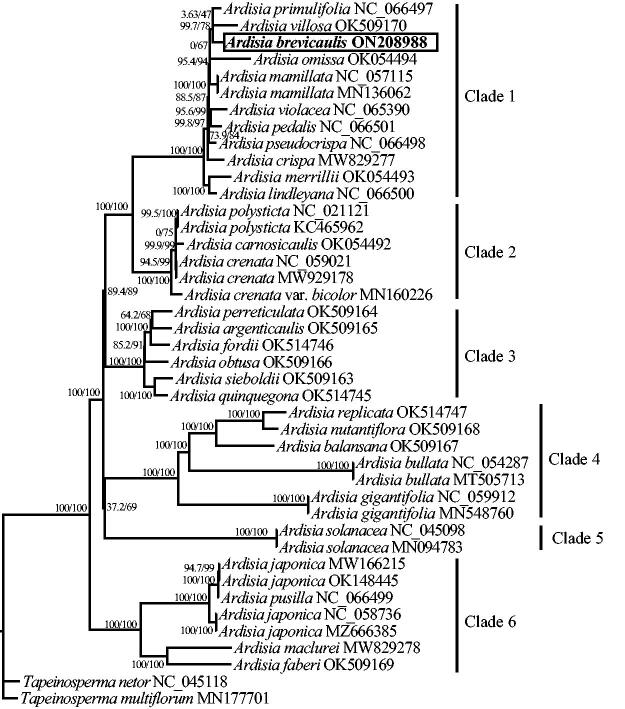
The maximum likelihood (ML) tree based on the complete chloroplast genome sequences of *Ardisia brevicaulis* and related species. Numbers in parentheses are SH-aLRT/UFBoot supports (%). The accession number of GenBank for each species is listed after species name.

The total length of the cp genome is 156,742 bp, with a total GC content of 37.1%. A typical quadripartite structure was shown in the cp genome, including a large single-copy (LSC) region (86,329 bp), a small single-copy (SSC) region (18,417 bp), and two inverted repeat regions (IRs; 25,998 bp). 134 genes (89 protein-coding, 37 tRNA, and 8 rRNA genes) were annotated in the whole cp genome, containing 115 unique genes (81 protein-coding, 30 tRNA, and 4 rRNA genes). Among them, 15 genes (*atp*F, *ndh*A, *ndh*B, *pet*B, *pet*D, *rpl*2, *rpl*16, *rpo*C1, *rps*16, *trn*A-UGC, *trn*G-UCC, *trn*I-GAU, *trn*K-UUU, *trn*L-UAA, *trn*V-UAC) contained one intron, while the *clp*P and *ycf*3 genes contained two introns. In addition, *rps*12 gene underwent trans-splicing (Figure 2). The phylogenetic tree in this study revealed that *A. brevicaulis* is nested in one well-supported clade (Clade1; 100/100%) with other 10 *Ardisia* species, in which *A. brevicaulis* is closely related to *A. primulifolia* and *A. villosa* (99.7/78%; Figure 3).

## Discussion and conclusion

*Ardisia* is the largest genus from the family Primulaceae and one of the largest tropical genera with an estimate of about 400 to 500 species mainly distributed in the tropical Asia, Americas, Australia, and the Pacific Islands (APG IV 2016; Chen and Pipoly 1996; Frodin 2004). Its classification has received considerable attention from botanists in decades, but mainly focused on morphological classification, and presented great uncertainties (Julius et al. 2021). Recently, a molecular phylogeny of *Ardisia* based on nrITS and three cpDNA regions suggested the monophyly of subgenera *Akosmos*, *Bladhia* and *Crispardisia*, and the non-monophyly of subgenera *Tinu*s and *Icacorea*, but cannot distinguish many species within *Ardisia* well (Ku and Hu 2014). Julius et al. (2021) explored a comprehensive phylogenetic relationships among *Ardisia* and related genera from 177 samples based on nrITS. It well indicate the non-monophyly of *Ardisia*, but clearly suggested the need for further study of the phylogenetic relationship within *Ardisia* because of low supporting rates among many *Ardisia* species. In this study, the phylogenetic relationship among surveyed *Ardisia* species was well resolved, showing six well-supported clades (Figure 3). Among them, *A. brevicaulis* and other 10 *Ardisia* species were nested in one clade with high supporting rates (100/100%), in which *A. brevicaulis* is closely related to *A. primulifolia* and *A. villosa* (Figure 3). These results probably implied that the chloroplast genomes can better resolve the interspecific relationship within *Ardisia*. Moreover, since medicinal plants within the same phylogenetic groups may have the same or similar therapeutic compounds/effects (Hao and Xiao 2020; Gong et al. 2022;), it is likely that the phylogenetic results in this study can also provide useful information for the exploration of new medicinal resources in *Ardisia*. However, it is worth noting that our phylogenetic tree was constructed only based on 30 *Ardisia* species (including one variety) because of few reports and releases of the chloroplast genomes of *Ardisia* in NCBI at present. Given the numerous species and wide distribution of *Ardisia*, in future studies, more chloroplast genomes and more species of *Ardisia* from all over the world are badly needed to be sequenced and studied to better explore the evolution and phylogenetic relationship within the genus.

## Data Availability

The data in this study is openly available in GenBank of NCBI at [https://www.ncbi.nlm.nih.gov] under the accession number ON208988. The associated BioProject, SRA, and Bio-Sample numbers involved in this study are PRJNA824992, SRR18700737, and SAMN27502973 respectively.
